# Acute pulmonary edema following appendectomy under general anesthesia: A case report

**DOI:** 10.1097/MD.0000000000047002

**Published:** 2026-01-02

**Authors:** Yang Qu, Deyuan Li, Shujin Li, Yan He

**Affiliations:** a Anesthesia and Surgery Center of the First People’s Hospital of Jintang County, Sichuan Province, China.

**Keywords:** case report, general anesthesia, appendectomy, acute pulmonary edema

## Abstract

**Rationale:**

This article describes a case of acute pulmonary edema in a young patient who underwent laparoscopic appendectomy under general anesthesia. Written informed consent was obtained from the patient.

**Patient concerns:**

A 25-year-old male patient underwent laparoscopic appendectomy under general anesthesia. Thirty minutes after endotracheal tube removal, the patient developed acute respiratory distress symptoms, became agitated, experienced decreased oxygen saturation, and produced pink, frothy sputum.

**Diagnoses:**

Both lungs were auscultated, and bilateral breath sounds were weakened. Clear, wet rales were heard, and chest computed tomography confirmed bilateral pulmonary edema.

**Interventions:**

High-flow oxygen therapy via face mask, corticosteroid treatment to control inflammation, diuretics to reduce pulmonary edema, and other symptomatic treatments were administered.

**Outcomes:**

The patient’s condition stabilized and he was discharged after 9 days with no long-term complications.

**Lessons:**

Although acute pulmonary edema after routine laparoscopic surgery in young patients is very rare, caution should be exercised in the early stage after removing the endotracheal tube. Meticulous perioperative fluid management, optimization of extubation techniques to prevent airway obstruction, rigorous postoperative monitoring, maintenance of high vigilance, and prompt intervention are essential to prevent serious consequences.

## 1. Introduction

Negative pressure pulmonary edema (NPPE) is a type of noncardiogenic pulmonary edema caused by an increase in the absolute values of pleural pressure and transpulmonary negative pressure resulting from acute upper airway obstruction and forced inhalation.^[1,2]^ One study reported that the incidence of NPPE in patients with acute upper airway obstruction is as high as 12%,^[3]^ while another study reported that the incidence in patients with laryngospasm was 3%.^[4]^

The underlying mechanism is as follows: When obstruction of the upper respiratory tract occurs,^[5]^ the respiratory muscles contract forcefully to maintain ventilation, causing a sharp rise in negative pleural pressure. This abnormal increase in transpulmonary pressure is the main driving force of injury. It damages the integrity of the alveolar–capillary membrane, manifested as injury to vascular endothelial cells and alveolar epithelial cells and an increase in permeability. Initially, only the intravascular fluid components leak out, causing high-pressure pulmonary edema. If the negative pressure persists or intensifies, it can lead to tearing of the microvascular structures, causing red blood cells to leak, and triggering diffuse alveolar hemorrhage. When this damage is combined with fluid overload or cardiogenic factors, pulmonary edema rapidly deteriorates.^[6–8]^ Pulmonary atrophy is common during the perioperative period; it reduces lung compliance, affects oxygenation efficiency,^[9]^ and exacerbates the movement of body fluids into the alveoli.^[7]^ Furthermore, the residual effects of anesthetic drugs may cause airway obstruction, creating a negative-pressure environment that can, in turn, trigger pulmonary edema.^[6]^ Postoperative pulmonary complications, such as pulmonary edema, have been extensively documented in young and healthy populations. However, acute episodes during conventional laparoscopic appendectomy are relatively rare and require close clinical attention.^[10,11]^

This case report describes acute pulmonary edema in a 25-year-old male following laparoscopic appendectomy under general anesthesia. It aims to highlight key risk factors in perioperative management through a detailed analysis of the clinical course, including the importance of precise liquid control, optimization of extubation techniques, and early monitoring interventions. This report not only enhances understanding of the mechanism of postanesthesia pulmonary edema in the existing literature,^[8,12]^ but also provides a warning for clinical practice, urging physicians to remain highly vigilant during common procedures such as appendectomy to prevent serious consequences.

## 2. Patient information

A 25-year-old man (height: 168 cm; weight: 55 kg) was admitted to the hospital because of pain around the navel that had persisted for 5 hours. He had no history of prior surgery or other diseases. Written informed consent was obtained from the patient, and the hospital ethics committee confirmed that this case report did not require ethical review.

## 3. Clinical findings and therapeutic interventions

**Figure 1.**
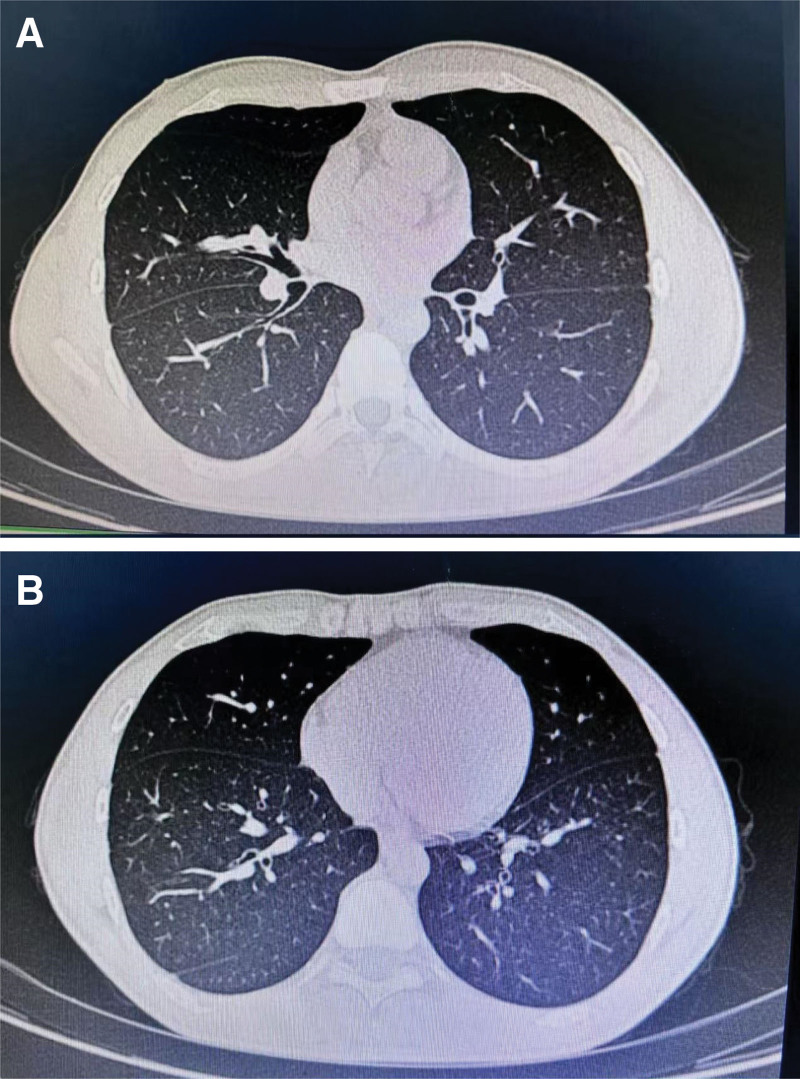
(A1, A2) Preoperative chest CT scans

The 12-lead electrocardiogram (ECG) was normal before surgery. A chest computed tomography (CT) scan revealed multiple nodules in both lungs, which were predominantly inflammatory in nature (Fig. 1A1, A2). The patient reported that he had pulmonary nodules several years ago that did not require special treatment. Preoperative laboratory evaluations indicated normal renal and hepatic function; the white blood cell count on routine testing was 19.9 × 10^9^/L, and the neutrophil percentage was 89.3%, consistent with acute appendicitis. A marked decrease in these values was observed after cauterization. The patient’s vital signs were stable, with a blood pressure of 116/82 mm Hg, a heart rate of 67 beats/min, and a body temperature of 36.8℃. Physical examination revealed no abnormalities.

**Figure 2.**
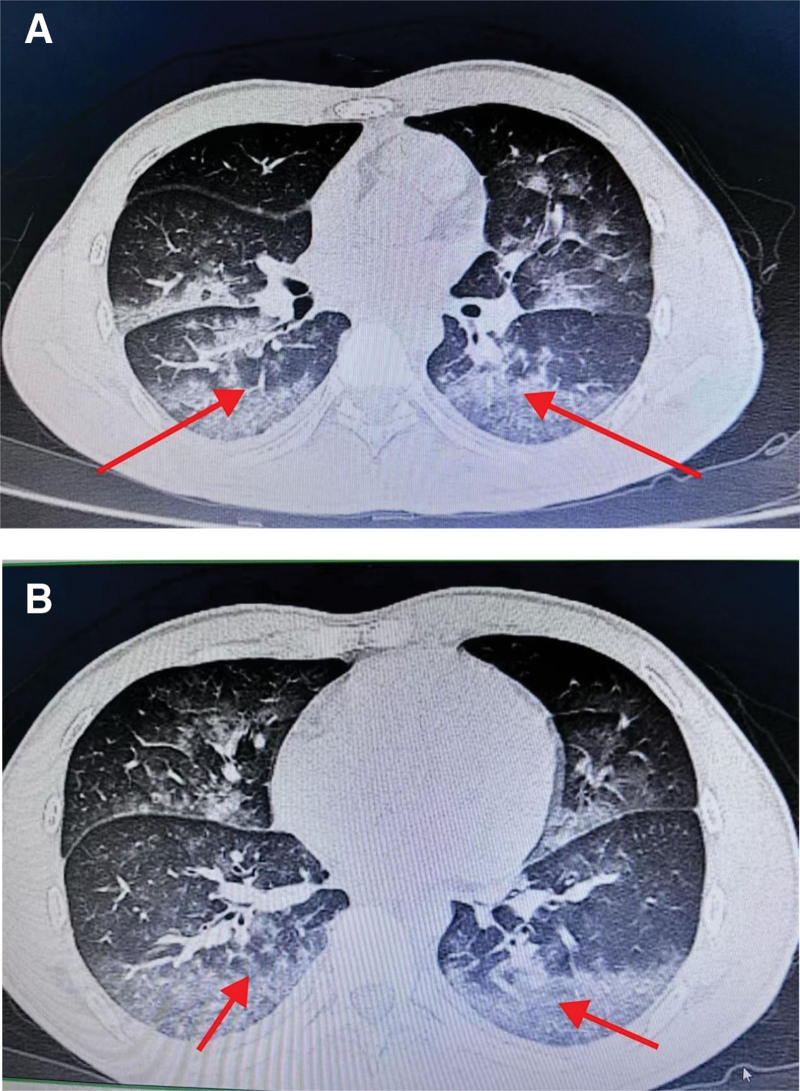
(B1, B2) Postoperative chest CT scans of the same area. CT = computed tomography.

Upon arrival in the operating room, routine monitoring revealed an initial blood pressure of 118/64 mm Hg, a heart rate of 88 beats/min, and an oxygen saturation of 98%. ECG findings were normal, except for mild tachycardia. General anesthesia was induced using midazolam (2.0 mg), propofol (100 mg), sufentanil (17.5 µg), and rocuronium bromide (60 mg). Endotracheal intubation was uneventful, and the airway was secured using a reinforced, cuffed tracheal tube (inner diameter 7.0 mm). Anesthesia was maintained with desflurane (1.5–2.0 minimum alveolar concentration) in 40% oxygen and a remifentanil infusion (0.1–0.2 µg/kg/min). Volume-controlled ventilation was set to a tidal volume of 6.0 mL/kg of predicted body weight, with a peak inspiratory pressure of ≤ 18 cmH₂O. The surgery lasted approximately 35 minutes (20:50–21:25) and was uneventful, with minimal blood loss and stable intraoperative hemodynamics. At 21:28, the patient regained spontaneous breathing, and neostigmine (1.0 mg), atropine (0.5 mg), and flumazenil (0.2 mg) were administered intravenously. The tracheal tube was removed at 21:30 after the patient’s tidal volume and respiratory rate reached the extubation index. During the operation, 500 mL of crystalloid solution was infused.

Thirty minutes later (22:00), the patient suddenly became agitated and restless; his oxygen saturation dropped to 66%, and his heart rate increased. Oxygen was immediately administered via face mask at 7.0 L/min. The oxygen saturation gradually increased to 92% and did not decrease again. For safety reasons, the anesthesiologist administered midazolam (1.0 mg) intravenously for sedation. Arterial blood gas analysis showed: pH 7.26, partial pressure of carbon dioxide (PaCO_2_) 52.9 mm Hg, partial pressure of oxygen (PaO_2_) 127 mm Hg, base excess (BE) -3, Na^+^ 142 mmol/L, and K^+^ 4.2 mmol/L. After 20 minutes of controlled breathing, the patient gradually regained consciousness and became less agitated; however, the oxygen saturation remained at 89% to 95%. Repeat arterial blood gas analysis revealed: pH 7.36, PaCO_2_ 40.9 mm Hg, and PaO_2_ 135 mm Hg. The patient experienced intermittent coughing with a small amount of pink, frothy sputum. Dexamethasone (10 mg) was administered intravenously. Auscultation of both lungs revealed coarse breath sounds and a few wet rales. As the patient was conscious and did not report any other discomfort, no emergency chest X-ray or CT examination was performed. The patient returned to the ward at 22:50 pm. The timeline of events is shown in Table 1.

**Table 1:** Timeline.

2025.6.13	20:05	The patient was admitted to the room. The blood pressure was 116/82 mm Hg, the heart rate was 67 beats/minute, the oxygen saturation was 98%, and the body temperature was 36.8℃. Auscultation of both lungs revealed clear and symmetrical breath sounds.
	20:10	Anesthesia induction and intubation were successful
	20:50	Surgery begins
	21:25	Surgery completed
	21:28	Restore spontaneous breathing and administer intravenous muscle relaxant antagonists
	21:30	Remove the endotracheal tube when the criteria for extubation are met
	22:00	The patient suddenly became agitated, with SpO_2_ dropping to 66%. Immediate action was taken: hands were placed under the jaw to open the airway, oxygen flow was increased, and 1 mg of midazolam was administered intravenously for sedation. SpO_2_ gradually rose to 92%.
	22:20	The patient regained consciousness, with oxygen saturation maintained at 89–95%, intermittent cough, and a small amount of pink sputum. Auscultation of both lungs showed coarse breath sounds and a small amount of wet rales.
	22:50	Return to ward

SpO_2_ = peripheral capillary oxygen saturation.

## 4. Follow-up and outcomes

After returning to the ward, the patient complained of breathing difficulties and produced pinkish sputum. The patient was administered high-flow oxygen via a mask and received symptomatic treatment. His oxygen saturation was maintained at 88% to 92%. Arterial blood gas analysis performed on postoperative day 1 showed: pH 7.34, PaCO_2_ 41.5 mm Hg, PaO_2_ 81.8 mm Hg, BE −3.8, Na^+^ 140.1 mmol/L, and K^+^ 3.75 mmol/L. A repeat blood gas analysis on postoperative day 3 showed: pH 7.37, PaCO_2_ 44.6 mm Hg, PaO_2_ 72.1 mm Hg, and BE 0.1. Chest CT reexamination (Fig. 2B1, B2) revealed multiple scattered patchy and ground-glass opacities in both lungs, most prominent in the dorsal segments, and a small amount of bilateral pleural effusion, suggesting pulmonary edema. The white blood cell count and percentage of neutrophils decreased. A multidisciplinary consultation was recommended for further diagnosis and treatment. After appropriate symptomatic treatment, the patient was discharged 9 days after surgery. At follow-up, he reported no discomfort or complications.

## 5. Discussion

NPPE may be triggered by various factors after general anesthesia, including mechanical ventilation–related lung injury, atelectasis, or negative-pressure effects caused by fluid redistribution or airway obstruction.^[13]^ Studies have shown that stress and strain during mechanical ventilation can damage lung tissue and increase the risk of postoperative pulmonary complications.

In this case, muscle relaxant antagonists were administered intravenously before the removal of the tracheal tube, and the tidal volume had reached 6 mL/kg before the tube was removed; thus, it can be concluded that there was no residual or very little residual muscle relaxant. However, the literature emphasizes that mechanical ventilation may cause alveolar overdistension or microdamage, leading to inflammation and pulmonary edema, even when the parameters are moderate.^[6]^ No “crowing” sounds were detected (excluding laryngospasm), and auscultation of both lungs revealed no wheezing (excluding bronchospasm). A dental pad was placed beside the patient’s tracheal tube, confirming no bite blockage. The patient was young and had no history of surgery or complications. The patient’s preoperative ECG was normal, and no significant ECG abnormalities were observed during the operation or after tube removal. The results of the postoperative ECGwhen the patient was returned to the ward were identical to those of the preoperative ECG (excluding cardiac causes). The patient weighed 55 kg and had fasted for more than 8 hours before surgery. The calculations showed a required fluid intake of 95 mL/hour (4 × 10 + 2 × 10 + 1 × 35), for a total of 760 mL. However, only 500 mL of crystalloid solution was administered during the procedure (excluding excessive fluid accumulation). No urinary catheter was placed owing to the short operation time; hence, urine output could not be recorded. Preoperative chest CT scan showed persistent inflammatory nodules with no significant changes over the years. The patient had no cough, sputum production, or fever prior to surgery. Auscultation revealed clear and symmetrical breath sounds in both lungs, ruling out pulmonary infection. When the patient’s oxygen saturation decreased, the jaw was immediately supported with both hands to open the airway, and oxygen with mask pressure was provided, and oxygen saturation gradually increased; therefore, we deduced that the airway was temporarily blocked by the tongue falling back after intubation. In summary, this event may be related to postoperative airway obstruction, which is highly consistent with the pathophysiology of NPPE. Upper airway obstruction, such as lingual drooping, leads to increased negative pressure in the chest cavity, promoting the exudation of pulmonary tissue fluid. Final imaging findings confirmed the diagnosis of NPPE.

A study reported 4 cases with similar findings, in which tongue retraction was the primary trigger.^[14]^ Another report also described laryngospasm following extubation that eventually progressed to NPPE.^[2]^ This underscores the critical role of airway obstruction in the pathogenesis of NPPE. In one case, a 54-year-old female patient underwent intramedullary pin fixation for an intertrochanteric femur fracture under general anesthesia. The patient had a history of depression and presented with sedation and apathy preoperatively and delayed emergence postoperatively. Upon removal of the laryngeal mask airway, a decreased level of consciousness was observed in the post-anesthesia care unit, along with a peripheral oxygen saturation (SpO_2_) level that decreased to 89%. The patient also expectorated pink, frothy sputum. After sustained oxygenation via a mask, dexamethasone and furosemide were administered intravenously, and the SpO_2_ level increased to 95%. The patient was then transferred to the intensive care unit (ICU). In another case, a 50-year-old male patient underwent debridement and tendon anastomosis for an open wound in the right calf under general anesthesia. The patient had a history of habitual snoring. Two minutes after extubation, the patient exhibited signs of tongue droop and experienced difficulty in ventilation. Oxygen was delivered via a mask with assisted ventilation using mandibular support, maintaining SpO_2_ levels above 80%, The patient coughed up pink, frothy sputum, and intravenous methylprednisolone (40 mg) was administered, which improved the SpO_2_ level to above 90%. The patient was subsequently admitted to the ICU. In the first case, the patient had a history of mental illness, and in the second case, the second patient had a history of snoring, and the duration of operation was nearly 2 hours. Meanwhile, in our case, the patient was young and had no history of disease, and the operation time was short; hence, we believe this case is rare and relevant.

This case demonstrates that preventing NPPE during the perioperative period is dependent on avoiding respiratory obstruction. For example, after the removal of the tracheal tube, an oropharyngeal airway can be placed to prevent the tongue from falling back; the patient should be observed at all times to detect abnormalities. In this case, high-flow oxygen therapy, dexamethasone, and diuretics were administered. Hyperbaric oxygen therapy can help relieve hypoxemia. Corticosteroids suppress inflammation, whereas diuretics reduce fluid overload. However, the literature states that acute pulmonary edema is characterized by damage to the pulmonary parenchyma, and the standard treatment remains supportive, as no targeted pharmacologic therapy has been proven effective.^[15]^ The literature emphasizes that “monitoring and anticipation” are the core of postoperative management,^[16]^ especially in cases of acute pulmonary edema, where reestablishing lung function depends on rapid intervention.^[17]^

## 6. Conclusion

NPPE following laparoscopic appendectomy under general anesthesia is rarely reported, highlighting the need for meticulous management during the perioperative period—especially for acute events in the early post-extubation phase, timely identification of respiratory obstruction types. This requires continuous monitoring of the patient’s condition. Even minor surgeries should not be performed lightly. The prevention and treatment of pulmonary edema after general anesthesia require a multidimensional approach, including optimizing mechanical ventilation parameters (e.g., avoiding high driving pressure), closely monitoring fluid balance, Preventing airway obstruction and maintaining a high level of vigilance for early identification.

## Acknowledgments

The authors appreciate the contributions of the medical teams involved in the patient’s care.

## Author contributions

**Conceptualization:** Yang Qu, Shujin Li, Yan He.

**Data curation:** Yan He.

**Methodology:** Yan He.

**Project administration:** Deyuan Li, Shujin Li.

**Supervision:** Shujin Li.

**Validation:** Yan He.

**Writing – original draft:** Yang Qu.

**Writing – review & editing:** Yang Qu, Deyuan Li.
